# Circular RNAs in Skeletal Muscle Aging and Sarcopenia: Mechanistic Evidence, Biomarker Potential, and Therapeutic Readiness

**DOI:** 10.3390/ijms27198844

**Published:** 2026-10-03

**Authors:** Thanh Huu Phan Ngo, Wan Lee

**Affiliations:** 1 Department of Biochemistry, Dongguk University College of Medicine, 123 Dongdae-ro, Gyeongju 38066, Republic of Korea; thngo139@gmail.com; 2 Medical Research Institute, Dongguk University College of Medicine, 123 Dongdae-ro, Gyeongju 38066, Republic of Korea

**Keywords:** circular RNA, skeletal muscle aging, sarcopenia, biomarkers, mechanotransduction, RNA therapeutics

## Abstract

Skeletal muscle aging arises from interacting defects in proteostasis, mitochondrial quality control, inflammatory signaling, neuromuscular integrity, regenerative capacity, and mechanical–metabolic plasticity, and may culminate in sarcopenia. Circular RNAs (circRNAs) are plausible regulators and biomarker candidates, but most evidence derives from developmental myogenesis, expression profiling, or non-aging atrophy models. This critical review organizes the literature around skeletal muscle aging and applies a four-level framework encompassing discovery, mechanistic support, in vivo causality, and functional intervention. We assess human linkage, species, treatment timing, locus of action, and endogenous or engineered modality separately. circSnd1 and circDdb1 currently provide the strongest combination of functional preclinical evidence and association with aged human skeletal muscle. circFUT10, circBBS9, circNEB, and circGLIS3 define less mature regenerative, discovery, histological, or muscle-quality domains. Non-aging atrophy studies, including circTmeff1 and engineered circmiR-29b, test whether proposed mechanisms remain active under defined catabolic stress. Human blood and serum-vesicle studies provide discovery-level associations with strength or mobility, but clinical validation of a circulating circRNA biomarker for sarcopenia remains insufficient. Therapeutic development is constrained by quantitative target credibility, systemic skeletal-muscle exposure, post-onset functional efficacy in aged organisms, safety, reversibility, and independent replication. The field should now prioritize rigorous validation over additional descriptive cataloging.

## 1. Introduction

Sarcopenia is not synonymous with low muscle mass. Contemporary European, Asian, and global consensus initiatives converge on a clinically important principle. Strength and function are central to the phenotype, whereas muscle quantity and quality provide complementary confirmation and biological context [1,2,3]. A molecular intervention that enlarges fibers without restoring force, power, endurance, or physical performance cannot by itself be assumed to correct sarcopenia. Skeletal muscle aging is better viewed as a progressive loss of adaptive reserve in a tissue that must continuously integrate nutritional, mechanical, neural, metabolic, and inflammatory signals [4,5,6].

This loss of reserve emerges from interacting processes. Aging alters protein turnover and anabolic responsiveness, mitochondrial quantity and quality, redox homeostasis, inflammatory tone, motor-unit connectivity, extracellular-matrix organization, satellite-cell behavior, and responses to mechanical and metabolic stress [4,5,6,7,8]. These domains are interdependent. Denervation modifies fiber composition and mitochondrial demand. Matrix remodeling changes force transmission and mechanosensing. Mitochondrial dysfunction can amplify redox and innate immune signaling, while regenerative impairment alters cellular composition. The network architecture helps explain why changes in a single biochemical readout often fail to predict functional recovery.

Circular RNAs (circRNAs) are attractive within this framework because they are structurally distinct RNA isoforms generated predominantly by back-splicing and can display tissue- and state-selective expression [9,10,11,12,13,14]. Selected circRNAs can interact with microRNAs (miRNAs) or RNA-binding proteins (RBPs), influence host-gene or transcriptional processes, and in some cases support translation [15,16,17]. Engineered circRNAs extend these properties into synthetic decoys and durable protein-expression platforms [18,19,20]. CircRNA abundance can also rise in aging tissues, although accumulation alone does not establish a functional role [21].

Previous reviews have addressed noncoding RNAs in muscle aging, circRNAs in musculoskeletal biology, circRNA–host–gene relationships in myogenesis, and circRNAs in muscle function and disease [22,23,24,25]. A recent review focused on circRNA-RBP mechanisms in exercise-induced skeletal-muscle remodeling and the associated mechanistic controversies [26]. The present review addresses a distinct translational question: how should aging specificity, human linkage, biomarker readiness, and therapeutic maturity be graded? Differential expression, developmental myogenesis, endogenous mechanism, functional rescue, and human association are often discussed together despite their different evidentiary meanings. This review therefore evaluates which circRNA claims meet progressively stricter tests of molecular identity, quantitative mechanism, aging relevance, in vivo function, human linkage, biomarker validation, and therapeutic feasibility.

Three questions organize the review. First, which circRNA mechanisms are supported directly in aging skeletal muscle or in models that meaningfully inform sarcopenia? Second, what evidence would be required before a tissue or circulating circRNA could be considered a biomarker of biological muscle aging instead of a generic disease-associated RNA? Third, which intervention concepts have progressed far enough to justify development in older organisms? Denervation, immobilization, glucocorticoid exposure, inflammation, and cancer cachexia are retained when they test the portability of an aging-relevant mechanism, but they are not treated as interchangeable evidence for sarcopenia.

## 2. Evidence Review Approach

### 2.1. Literature Identification, Scope, and Retraction Audit

This article is a structured critical narrative review, not a PRISMA-registered systematic review. The original English-language search was updated through 12 September 2026. Table 1 summarizes the sources and search concepts. Targeted revision searches and source checks were conducted on 27 September 2026 for blood biomarkers, cellular senescence, endocrine signaling, and RBP mechanisms. These updates do not constitute a new systematic search of every topic or a repeat of the registry search.

Citation-linked searches, publisher records, and available primary full texts were used to resolve candidate-specific claims. Candidate assignments were checked against the Level 1–4 criteria and reconciled across the text and tables. Reviews supplied context and candidate leads but did not substitute for primary mechanistic evidence. The original retraction audit and registry search date was 12 September 2026. This synthesis does not claim exhaustive coverage, and non-retrieval is not evidence of biological absence.

Primary studies were prioritized for mechanistic and readiness judgments. Reviews and consensus documents were used for clinical definitions, field context, analytical standards, and candidate discovery, but not as substitutes for primary evidence. The aging/sarcopenia evidence pool included naturally aged animals, aged human skeletal muscle, aged skeletal-muscle stem cells, aging-associated muscle quality, or exercise modulation of aged muscle. A comparator wasting pool included denervation, unloading or immobilization, glucocorticoid exposure, inflammatory atrophy, and cancer cachexia. Developmental or livestock myogenesis studies were retained only when they established a mechanism with a plausible connection to aging biology, and when their species and developmental context were explicitly stated.

Human linkage was assigned only when a study included directly relevant human skeletal muscle, human myogenic cells, or a clinically relevant human biospecimen. Sequence conservation alone was not counted as human evidence. For human tissue studies, expression association was distinguished from mechanistic or interventional validation.

Editorial status was checked independently of citation frequency. Publisher records confirmed that three reports that could otherwise have entered muscle-circRNA narratives had been retracted. These reports concern gastric cancer exosomal ciRS-133, circ-FoxO3 during myoblast differentiation, and the 2018 bovine circFUT10 and miR-133a study. All three were excluded from the positive evidence synthesis. The last report differs from the separate 2021 study of circFUT10 in aged skeletal muscle stem cells [27]. Table 2 records how each case was handled.

### 2.2. Evidence Levels and Independent Modifiers

Four evidence levels are used. Level 1 includes differential expression, profiling, network prediction, exercise responsiveness, or clinical association without candidate-level causal validation. Level 2 requires validated circular identity, candidate perturbation, and a directly tested molecular interaction or pathway, but lacks decisive in vivo evidence in aging or wasting. Level 3 requires circRNA manipulation that changes an aging- or wasting-relevant tissue phenotype in vivo. Level 4 requires an in vivo intervention with a meaningful functional endpoint such as grip, force, contractility, endurance, or power and concordant tissue evidence. Post-onset treatment is recorded separately as an intervention-timing modifier.

The evidence level is separated from five modifiers. These are human linkage, species, intervention timing, anatomical locus of action, and modality. Human linkage is classified as none, human cells, human tissue association, or a clinically phenotyped human cohort. Intervention timing is preventive or post-onset. The locus of action is muscle-intrinsic or systemic and indirect, while the modality is an endogenous target or an engineered circRNA. This separation prevents a small observational human cohort from automatically outranking a rigorous functional animal intervention and prevents non-mammalian evidence from being interpreted as equivalent to human readiness. The evidence levels are summarized in Table 3. These levels are an interpretive framework developed for this review rather than a validated risk-of-bias instrument.

## 3. Biological Architecture of Skeletal Muscle Aging

### 3.1. Clinical Phenotype, Proteostasis, and Mitochondrial Function

The European Working Group on Sarcopenia in Older People 2 (EWGSOP2), Asian Working Group for Sarcopenia (AWGS) 2019, and Global Leadership Initiative in Sarcopenia (GLIS) differ in operational details but converge on the concept that sarcopenia is a functional skeletal-muscle disorder rather than a radiologic diagnosis based solely on lean mass [1,2,3]. Molecular studies should connect candidate pathways to strength or performance whenever possible. Muscle quality is equally important. Fibrosis, fat infiltration, denervation, fiber-type remodeling, mitochondrial deficiency, and altered specific force can produce markedly different function at similar muscle quantities. Consensus work on biomarkers of skeletal-muscle aging likewise emphasizes multidimensional assessment and linkage of molecular measures to tissue structure and function [28].

Muscle mass reflects the integrated balance of protein synthesis, degradation, and quality control. The ubiquitin-proteasome and autophagy-lysosome systems are required for normal turnover, but chronic imbalance can lead to the loss of contractile proteins and organelles [4,29]. Aging can also reduce responsiveness to nutritional, insulin, or mechanical stimuli, a concept commonly described as anabolic resistance. Importantly, the human literature is not uniform. Blunted protein-anabolic responses have been demonstrated in older adults under some insulin and exercise conditions [30,31], whereas adequate provision of essential amino acids can elicit preserved responses in healthy older adults [32]. Thus, anabolic resistance is better treated as context- and dose-dependent than as an invariant property of chronological aging.

This nuance matters for circRNA studies. Changes in atrogin-1, MuRF1, phosphorylated mechanistic target of rapamycin (mTOR) pathway proteins, or a single translation marker can support a mechanism but do not demonstrate restoration of protein homeostasis. Stronger studies should connect the molecular pathway to integrated protein turnover, myofibrillar integrity, and force-generating capacity. Human and experimental studies of SESN1 and the HSF1-SIRT3-PGC-1alpha axis illustrate how stress-response pathways can influence proteostasis, mitochondria, and resilience [33,34].

Mitochondrial abnormalities are consistently associated with aging skeletal muscle, including changes in biogenesis, dynamics, mitophagy, respiration, and redox regulation [7,35]. Chronological aging and physical inactivity, however, are difficult to separate. Deeply phenotyped human studies show that exercise status materially modifies mitochondrial capacity and physical function [36], and three-dimensional analyses reveal age-associated remodeling of mitochondrial architecture [37]. CircRNA studies that invoke mitochondrial mechanisms should move beyond adenosine triphosphate (ATP) content or a single respiratory-complex protein and test mitochondrial quality control, respiration, or substrate use together with muscle function.

### 3.2. Inflammation, Regeneration, and Neuromuscular Remodeling

Chronic low-grade inflammation and oxidative stress can reinforce catabolism, mitochondrial damage, insulin resistance, and regenerative dysfunction [5,8]. Inflammatory markers, however, are not specific to aging muscle. Tumor-derived factors, adipose signals, immune-cell products, vascular changes, and local myofiber signaling can converge on nuclear factor kappa B (NF-kappaB), Janus kinase-signal transducer and activator of transcription (JAK-STAT), cyclic GMP-AMP synthase-stimulator of interferon genes (cGAS-STING), and related stress pathways. For extracellular-vesicle or circulating circRNA studies, source attribution is a central biological requirement.

Aging changes satellite-cell number, quiescence, self-renewal, and regenerative competence, but regenerative failure is not identical to basal sarcopenia. Geriatric muscle stem cells can transition toward senescence-like states, and experimental rejuvenation can improve regenerative function and post-injury strength [38,39]. Conversely, inducible satellite-cell depletion can severely impair regeneration without necessarily accelerating basal sarcopenia in sedentary adult mice [40]. A circRNA that alters myoblast or satellite-cell proliferation should not be labeled a sarcopenia regulator unless the mechanism is connected to maintenance or recovery of aged muscle in vivo.

**Figure 1:**
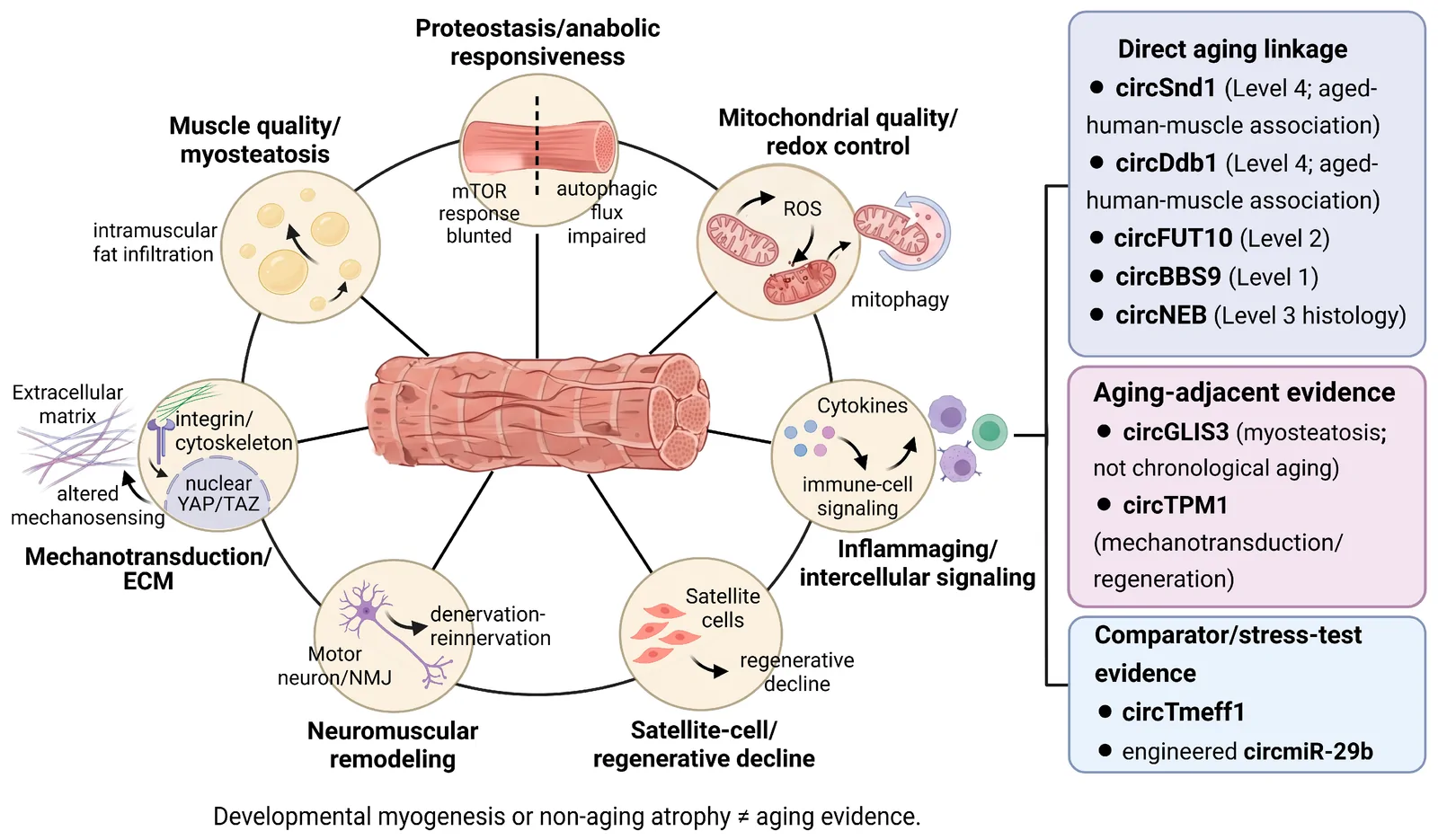
Biological architecture of skeletal muscle aging and the position of current circRNA evidence. Aging and reduced physical activity converge on interconnected domains, including proteostasis and anabolic responsiveness, mitochondrial quality and redox control, inflammation, regenerative decline, neuromuscular remodeling, mechanotransduction and extracellular matrix change, and myosteatotic remodeling. We separate candidates with direct aging linkage from aging-adjacent evidence and comparator or stress-test evidence. circSnd1 and circDdb1 each combine Level 4 functional preclinical evidence with an aged-human-muscle association; these evidence dimensions remain independent, and the associations do not constitute clinical validation. circFUT10, circBBS9, and circNEB occupy lower preclinical evidence levels. The proteostasis module depicts context-dependent anabolic resistance and dysregulated autophagic flux. circGLIS3 and circTPM1 are classified as aging-adjacent, whereas circTmeff1 and engineered circmiR-29b are comparator or stress-test evidence. Arrows depict illustrative processes and connections, not circRNA causality; colored boxes identify the labeled evidence categories. The figure is a selective map of muscle-linked evidence, not an exhaustive list of circRNAs regulating cellular senescence. Candidates affecting autophagy, mitophagy, or inflammation in other tissues remain hypotheses for aging-muscle studies. Developmental myogenesis and non-aging atrophy are not interchangeable with aging evidence. circRNA, circular RNA; ECM, extracellular matrix; mTOR, mechanistic target of rapamycin; NMJ, neuromuscular junction; ROS, reactive oxygen species; YAP/TAZ, Yes-associated protein and transcriptional coactivator with PDZ-binding motif. Created in BioRender. Lee, W. (2026) https://BioRender.com/osvo17l, accessed on 12 September 2026.

CircRNAs implicated in cellular senescence remain plausible candidates even when human-muscle data are unavailable. In human WI-38 fibroblasts, circPVT1 depletion promoted senescence, with evidence implicating let-7 regulation [41]. This establishes a non-muscle cellular mechanism, not an effect on sarcopenia. CircRNAs linked to autophagy, mitophagy, or inflammatory signaling in other tissues can likewise motivate muscle studies, but shared pathway labels do not establish tissue transferability. Candidate prioritization should test circular identity and endogenous perturbation in aged myofibers or their supporting cells, followed by tissue and functional outcomes. Figure 1 therefore depicts selected muscle-linked evidence, not a complete inventory of senescence-associated circRNAs.

Neuromuscular aging includes motor-unit loss, repeated denervation-reinnervation, fiber-type grouping, and changes in neuromuscular-junction stability [42,43,44]. Human biopsy studies have identified molecular signatures of denervation in older skeletal muscle [45]. More importantly, recent work across stages of primary human sarcopenia demonstrates progressive neuromuscular impairment using morphological, electrophysiological, and molecular measures [46], while the Study of Muscle, Mobility, and Aging (SOMMA) cohort links higher expression of denervation-responsive genes to lower muscle volume and poorer performance traits [47]. These human data justify retaining denervation models as mechanistic comparators, but an experimentally denervated young muscle remains a stress model and not a surrogate for the full biology of aging.

### 3.3. Mechanotransduction and Muscle Quality

Aged muscle receives and interprets mechanical signals in a changing physical environment. Physical activity, extracellular matrix composition, cytoskeletal organization, nuclear architecture, and mechanosensitive signaling all change with age. The linker of nucleoskeleton and cytoskeleton (LINC) complex, nuclear lamina, actin cytoskeleton, focal adhesions, and Yes-associated protein/transcriptional coactivator with PDZ-binding motif (YAP/TAZ) provide routes by which force is coupled to transcription, metabolism, and growth [48,49,50,51,52]. In a sarcopenia model, mechanical stimulation has been linked to YAP-mediated transcription of the carnitine transporter OCTN2 [53]. YAP also regulates skeletal-muscle substrate metabolism and can couple altered mechanical state to inflammatory aging pathways in other stromal contexts [54,55]. These observations justify direct tests of exercise-responsive circRNAs in aged muscle, although current evidence remains preliminary.

Myosteatosis provides a complementary muscle-quality axis. Imaging studies in older populations show that muscle density and intramuscular adipose tissue carry information beyond area alone and associate with adverse clinical or functional outcomes [56,57]. CircGLIS3 is relevant to this domain because its manipulation reduced high-fat-diet-associated skeletal-muscle fat infiltration in mice [58]. The model is metabolically relevant but does not represent chronological aging or sarcopenia. CircGLIS3 is classified below as aging-adjacent muscle-quality evidence and is not assigned direct aging linkage. Figure 1 places these candidates within the broader biological architecture of skeletal muscle aging.

### 3.4. Endocrine and Secreted-Factor Mechanisms

Endocrine regulation provides an additional route through which circRNAs could influence muscle aging indirectly. Hormones produced outside muscle must be distinguished from muscle-derived myokines and local secreted factors. A circRNA-induced change in receptor signaling within myocytes does not itself demonstrate altered hormone secretion. Insulin responsiveness is relevant to muscle anabolism [30], but both hormone availability and the response of aged muscle need to be measured.

A mechanistic precedent comes from pancreatic islets. The insulin-gene circular intronic RNA ci-Ins2, and its human counterpart ci-INS, support insulin secretion through a mechanism involving transactive response DNA-binding protein 43 (TDP-43) [59]. This establishes circRNA control of endocrine secretion outside muscle. It does not demonstrate that this pathway causes or treats sarcopenia. Within a distinct disease context, tumor circANAPC7 reduces transforming growth factor beta (TGF-beta) secretion and muscle wasting in pancreatic-cancer models [60]. This supports a secreted-factor route to muscle effects, but tumor-derived TGF-beta is not a muscle-secreted hormone and cancer cachexia is not primary sarcopenia.

A muscle endocrine mechanism would require circRNA perturbation in the proposed source cell, measurement of secreted protein and circulating exposure, and a response in the target tissue. Candidate myokines such as myostatin or interleukin-6 (IL-6) should be treated as hypotheses unless this causal chain is demonstrated. Conditioned-medium transfer, receptor blockade, and factor add-back could distinguish secretion-dependent effects from direct actions of the circRNA. In aged animals, the experiment should also separate changes in food intake, systemic metabolism, and activity from muscle protein turnover and contractile function.

## 4. circRNA Biology Relevant to Aging Muscle

### 4.1. Circular Identity and Molecular Mechanisms

Back-splicing creates a junction absent from the collinear host transcript. Candidate studies should establish the back-splice junction with orthogonal evidence, typically junction-spanning divergent reverse-transcription polymerase chain reaction (RT-PCR), sequence confirmation, and appropriate assessment of exonuclease resistance or transcript stability. Ribonuclease R resistance is supportive but not absolutely specific [10,11,12,13,14]. Localization should be measured when the proposed mechanism depends on access to nuclear, cytoplasmic, ribosomal, or extracellular compartments.

These experiments prove that a circular species exists, but they do not establish causality. Perturbation must distinguish the circular transcript from the host gene and linear isoforms. Junction-targeting antisense reagents, multiple independent loss-of-function reagents, circularization-deficient controls, and rescue with a perturbation-resistant circle provide substantially stronger causal evidence than generic overexpression. Coding claims additionally require direct identification of the back-splice-dependent product and separation of RNA-mediated effects from those of the encoded protein.

The demonstration that selected natural circRNAs can act as potent miRNA sponges established an important mechanism [15], but it also encouraged overgeneralization. Quantitative studies of competing endogenous RNA (ceRNA) interactions show that competition depends on the abundance of the competitor, the miRNA, and the accessible target pool [61]. A predicted site, reciprocal expression, and even a wild-type reporter can establish binding compatibility without demonstrating that endogenous circRNA abundance materially alters miRNA activity.

A convincing circRNA-ceRNA claim should integrate junction-specific perturbation, direct physical interaction, seed-dependent controls, endogenous target responses, and rescue or epistasis. Copy-number estimates, or at minimum abundance measurements relative to the implicated microRNA and target pool, substantially increase credibility. The requirement is especially important in aging muscle, where whole-tissue measurements average across myofibers, satellite cells, fibro-adipogenic progenitors, endothelial cells, immune cells, and neural structures.

RNA-binding-protein-centered and translation mechanisms deserve particular attention because several of the strongest muscle-wasting circRNA studies do not rely on classical miRNA sponging. Translation of endogenous circRNAs has been established in myogenic and other systems [16,17], and circRNA production can interact with canonical splicing decisions [14]. Coding claims should demonstrate the unique peptide or protein and its functional dependence on circularization. RBP mechanisms require direct binding and perturbational linkage between the complex and phenotype. These standards also reduce the risk of attributing host-gene effects to a nearby circular isoform.

### 4.2. RNA-Binding Proteins in Muscle circRNA Regulation

RBP interactions can regulate circRNA production, the activity of the circular transcript, or downstream mRNA fate. These roles should be distinguished from effects of a protein encoded by a circRNA. Ikeda et al. outline how circRNAs intersect with miRNA-loaded silencing complexes and other RNA-regulatory proteins in non-muscle diseases [62]. That framework is useful here, but its individual disease mechanisms cannot be assumed to operate in sarcopenia.

In muscle models, eukaryotic translation initiation factor 4A3 (EIF4A3) acts upstream of circSnd1 and circDdb1 expression [63,64]. This biogenesis role differs from a direct effector interaction. CircTmeff1 associates with TDP-43 and promotes its mitochondrial accumulation in experimental atrophy [65]. The study also reports a translated product, so the RNA-binding and protein-coding contributions must be evaluated separately. These findings support RBP-linked mechanisms in muscle wasting, with the model and human linkage assessed independently.

A complementary example is circSamd4 in differentiating C2C12 cells. Affinity purification and RNA immunoprecipitation identified interactions with purine-rich element-binding proteins A and B (PURA and PURB). CircSamd4 relieved their repression of myosin-heavy-chain transcription [66]. This is a circRNA–protein–transcription mechanism, not evidence that every circRNA acts through miRNA competition. It provides a myogenic precedent, while its relevance to aged muscle remains untested in that study. AU-rich element RNA-binding protein 1 (AUF1, encoded by HNRNPD) has more direct muscle-maintenance evidence. Auf1-deficient mice developed muscle wasting and impaired regeneration, with excessive matrix metalloproteinase 9 (MMP9) expression linked to altered mRNA decay [67]. That study does not establish a circRNA–AUF1 mechanism.

Within a proposed circRNA–miRNA–mRNA axis, Argonaute 2 (AGO2) links miRNA recognition to the silencing machinery. AGO2 enrichment can support complex association, but cannot establish that a circRNA sequesters enough miRNA to regulate endogenous targets. CircRNA and miRNA abundance, shared localization, target occupancy, and interaction-specific rescue remain necessary tests [15,61]. For a muscle RBP candidate, binding-site disruption should alter the relevant mRNA or protein output while preserving the linear host transcript. Rescue with an interaction-defective RBP or circRNA can then test whether the complex is required for the phenotype. These experiments distinguish a plausible regulatory connection from a demonstrated mechanism in aging muscle.

### 4.3. Extracellular circRNAs and Biomarker Interpretation

**Figure 2:**
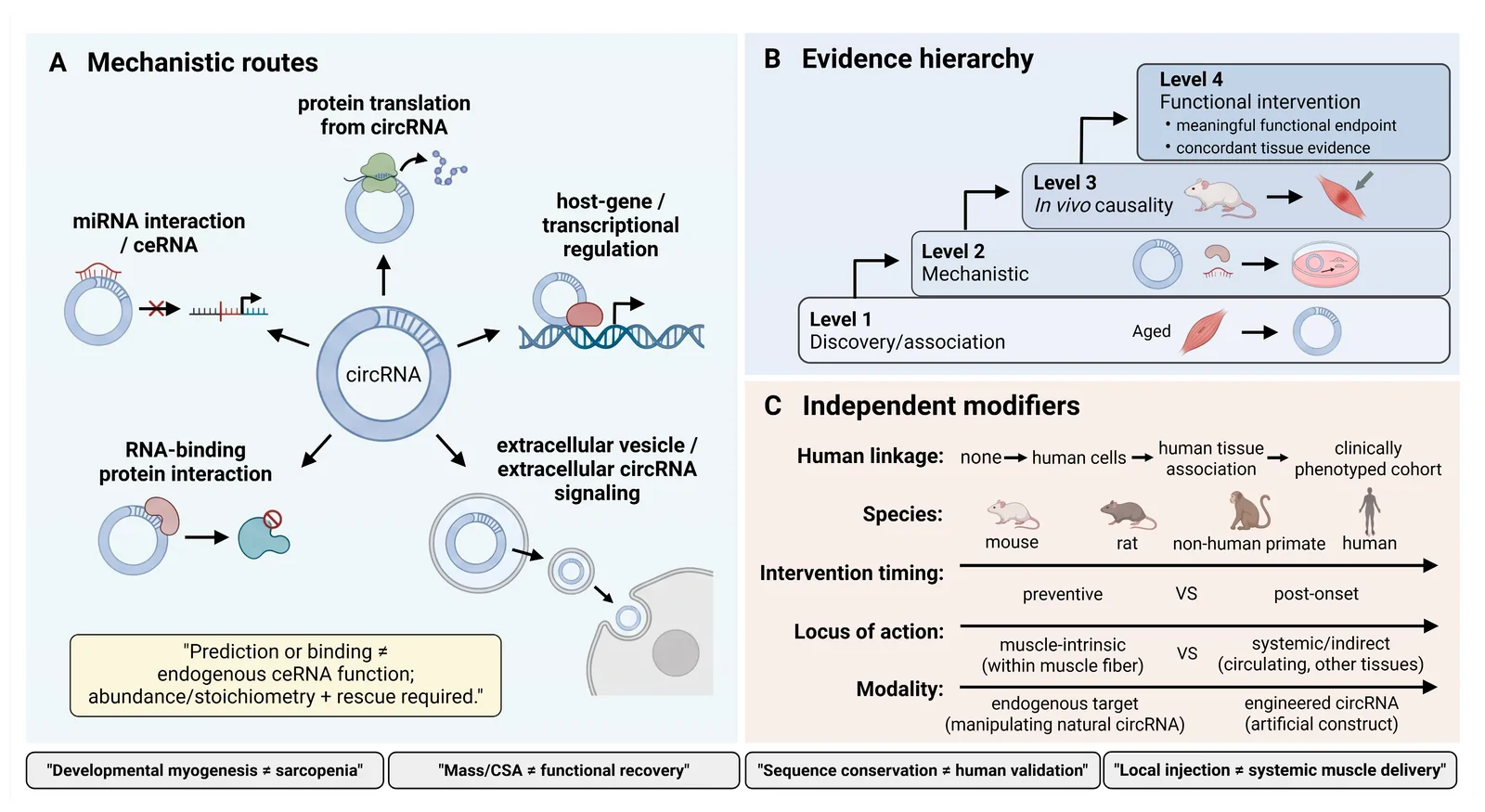
Mechanisms, preclinical evidence levels, and independent modifiers for circRNA studies in aging muscle. Panel (**A**) shows miRNA interaction, RBP interaction, translation, host-gene or transcriptional effects, and extracellular-vesicle signaling. Prediction or binding alone does not establish endogenous ceRNA function; quantitative abundance, stoichiometry, and rescue or epistasis are required. Panel (**B**) distinguishes Level 1 discovery or association, Level 2 mechanism, Level 3 in vivo causality, and Level 4 intervention with a meaningful functional endpoint and concordant tissue evidence. Arrows in panel A suggest mechanistic routes; arrows in panel C organize descriptors, not causal paths. Panel colors separate content without quantitative meaning. The staircase arrows denote increasing evidentiary thresholds and do not indicate biological causality. Panel (**C**) records human linkage, species and model, intervention timing, locus of action, and endogenous or engineered modality separately from the preclinical evidence level. Preventive treatment occurs before phenotype onset, whereas post-onset treatment begins after phenotype establishment. Bottom warnings distinguish developmental myogenesis from sarcopenia, mass or CSA from functional recovery, sequence conservation from human validation, and local injection from systemic muscle delivery. ceRNA, competing endogenous RNA; circRNA, circular RNA; CSA, cross-sectional area; miRNA, microRNA; RBP, RNA-binding protein. Created in BioRender. Lee, W. (2026) https://BioRender.com/jb40v4z, accessed on 12 September 2026.

Circular topology can increase RNA stability, but stability is not synonymous with tissue specificity, analytical robustness, or diagnostic value. Hemolysis can distort extracellular RNA measurements, while circRNA studies show marked enrichment in platelets and selective release in platelet-derived extracellular vesicles [68,69,70]. Residual cells and platelets can therefore alter the apparent circulating circRNA profile. These findings do not establish a universal pre-analytical protocol, but they show why hematologic source control and prospective validation of sample handling are essential. A circulating circRNA may originate from hematologic cells, liver, tumor, adipose tissue, or damaged muscle. Source attribution and a prespecified clinical phenotype are required before a blood signal is called a skeletal-muscle-aging biomarker. Figure 2 summarizes the mechanistic routes, evidence levels, and independent study modifiers.

## 5. circRNA Evidence Relevant to Skeletal Muscle Aging

### 5.1. Functional Benchmarks with Human Aging Linkage

circSnd1 currently provides one of the most complete experimental programs linking a circRNA to muscle atrophy and aging [63]. The study reported increased circSnd1 in a small human skeletal-muscle cohort of older individuals and examined human myotubes, mice, and C2C12 cells. EIF4A3-associated circSnd1 biogenesis was linked to an interaction between FAT10 (ubiquitin D) and eukaryotic translation elongation factor 1 alpha 1 (EEF1A1). This interaction reduced EEF1A1 ubiquitination and stabilized the protein. The program extended across denervation, immobilization, angiotensin II, dexamethasone, and tumor necrosis factor alpha-associated atrophy settings.

Its strength lies in the combination of structural validation, gain-and-loss experiments, tissue phenotypes, and functional endpoints, including grip and contractile measurements. The reported human comparison included five samples per age group. It was an expression association without clinically adjudicated sarcopenia, longitudinal outcomes, or independent validation. circSnd1 therefore meets Level 4 functional preclinical evidence but not clinical validation. Larger cohorts linked to strength and physical performance are needed.

circDdb1 provides a second high-level example [64]. It is increased in aged human skeletal muscle and multiple experimental atrophy conditions and encodes circDdb1-867aa. The encoded product interacts with eEF2 and increases inhibitory eEF2 Thr56 phosphorylation, directly linking circular RNA expression to reduced translational capacity. In vivo experiments included muscle size, grip strength, exercise capacity, and contractile force.

The mechanism is relevant to aging because it links a circRNA to protein synthesis at the elongation level. The reported human comparison used five young and five aged muscle samples obtained near the knee during arthroscopy. It did not include an accepted sarcopenia phenotype, longitudinal outcomes, or independent validation. circDdb1 meets Level 4 functional preclinical evidence and is associated with aged human skeletal muscle, but it has not been clinically validated.

Age-associated elevation of a circRNA does not by itself establish regulated induction. In post-mitotic muscle, increased RNA stability, reduced turnover, or altered cellular composition may produce apparent accumulation. Distinguishing regulated induction from passive accumulation requires circle-to-linear host-transcript ratios, junction-specific absolute quantification, cell-type resolution, and control for activity or disuse. These caveats particularly limit interpretation of the older-human-muscle associations for circSnd1 and circDdb1, although they do not negate the functional preclinical findings [10,11,12,13,14,21].

### 5.2. Regenerative and Exercise-Responsive Candidates

The 2021 circFUT10 study examined aged skeletal muscle stem cells and linked circFUT10 to a miR-365a-3p-HOXA9 mechanism that affects proliferation and differentiation [27]. This provides direct evidence in an aging-related regenerative compartment but does not demonstrate recovery of muscle mass, strength, or performance in an aged organism. The study supports Level 2 mechanistic evidence for aging. Citation identity is important because the earlier 2018 bovine circFUT10-miR-133a myoblast report was retracted in 2026. That retraction does not apply to the separate 2021 aged-stem-cell study.

Guo et al. identified circBBS9 as lower in aged mouse muscle and responsive to aerobic exercise [71]. The study also overexpressed circBBS9 in differentiated C2C12 myotubes. This reduced *Foxo3* and Atrogin mRNA and increased Pgc1α, Mfn1, and Atpase mRNA. These cellular perturbation data extend beyond profiling, but the readouts are transcript abundance, not mitochondrial flux or muscle function. The predicted miRNA network was not established by interaction-specific rescue, and candidate-directed efficacy in aged animals was not demonstrated. Under the present criteria, circBBS9 remains Level 1 with preliminary cellular perturbation support and an unresolved mechanism.

### 5.3. Aging Histology and Muscle Quality

circNEB is an instructive protein-coding example with in vivo aging data [72]. Multi-omics analysis identified a circNEB-derived peptide and linked it to SKP1-related control of p27-Kip1 and myogenic proliferation. Four 40-month-old New Zealand white rabbits received paired local plasmid injections in the hindlimbs. Short-term histological measures included muscle-fiber spacing and average fiber area. Grip, force, endurance, and other whole-animal functional outcomes were not reported. The evidence supports Level 3 in vivo causality with aged-organism histology but not a functional sarcopenia intervention.

circGLIS3 inhibits intramuscular adipogenesis and reduces skeletal-muscle fat infiltration in a high-fat-diet mouse model [58]. This provides Level 3 in vivo causal evidence for a muscle-quality phenotype. The biology is relevant because myosteatosis contributes to impaired muscle quality in older adults [56,57]. The study did not examine chronological aging, clinically defined sarcopenia, or functional recovery in old animals. circGLIS3 is therefore classified as aging-adjacent evidence that warrants direct testing in aged muscle. Table 4 compares the candidates with direct aging linkage.

## 6. Evidence from Non-Aging Atrophy Models

Aging is slow and heterogeneous and is accompanied by changes in activity, innervation, endocrine state, metabolism, and multimorbidity. Acute denervation, immobilization, glucocorticoid exposure, and inflammatory models simplify this complexity but remain experimentally useful. They can show whether a circRNA mechanism remains active under a defined catabolic stress and can support therapeutic timing experiments that are difficult in aging cohorts. Evidence from these models still requires confirmation in naturally old muscle.

### 6.1. Functional Stress-Test Studies

circTmeff1 is among the strongest comparator atrophy studies [65]. The work combined structural validation, gain- and loss-of-function experiments, multiple wasting conditions, a transactive response DNA-binding protein 43 (TDP-43) interaction mechanism, mitochondrial TDP-43 aggregation, mitochondrial DNA release, cGAS-STING signaling, and an encoded TMEFF1-339aa product. Suppression was tested after atrophy was established, and functional outcomes were measured. These features make circTmeff1 Level 4 comparator evidence and a strong stress test for mitochondrial and innate-immune pathways that are plausible in aging. The study does not establish a direct role in chronological skeletal-muscle aging or human sarcopenia.

Engineered circmiR-29b contains multiple imperfect miR-29b-binding sites and functions as a synthetic circular decoy [73]. It attenuated tissue-level atrophy across several contexts and was tested after immobilization-associated atrophy had developed. No force, grip, endurance, or power endpoint was reported. The study is therefore classified as Level 3 in vivo evidence with a post-onset timing modifier, rather than Level 4. It does not show that an endogenous circRNA naturally controls aging through the same design logic. In vivo expression was vector-mediated, meaning that DNA encoding a circularizing RNA precursor was delivered rather than a purified circRNA molecule. This distinction affects exposure, reversibility, redosing, and safety.

### 6.2. Systemic, Species-Limited, and Discovery Comparators

circANAPC7 reduces muscle wasting in pancreatic-cancer models through a tumor-centered PH domain leucine-rich repeat protein phosphatase 2 (PHLPP2)-AKT-TGF-beta mechanism [60]. The muscle benefit is biologically meaningful but indirect from the standpoint of skeletal muscle and should not be grouped with muscle-intrinsic aging targets. circPTK2 provides a complementary example of cachexia, centered on adipose lipolysis and adipogenesis [74]. circAGO3 provides in vivo inflammatory-atrophy evidence in chicken through a miR-34b-5p-TRAF3-NF-kappaB axis, whereas circCCDC91 links miR-15-family regulation to IRS1/IGF1-PI3K-AKT signaling predominantly in chicken myogenic systems [75,76]. These studies demonstrate mechanistic possibilities but have important species or anatomical limitations for translation.

Profiling of denervated rat muscle identified hundreds of differentially expressed circRNAs, and circSMOX has been associated with dexamethasone-treated C2C12 cells and amyotrophic lateral sclerosis (ALS)-related muscle contexts [77,78]. These resources are useful for discovery but do not establish causal drivers. circAtxn10 was identified in human skeletal-muscle myogenic cells and was reported to regulate C2C12 differentiation through a proposed miR-143-3p–CHRNA1 axis. The study did not establish quantitative endogenous ceRNA competition or test aging [79]. The TPM1 super-enhancer study linked circTPM1 to myogenic differentiation and regeneration through MYH10 binding and MYL3-dependent actomyosin assembly [80]. It provides Level 2 mechanistic evidence but did not test chronological aging, sarcopenia, or a circRNA-specific in vivo intervention. Table 5 summarizes the comparator and aging-adjacent studies.

## 7. Readiness of circRNAs as Biomarkers of Skeletal Muscle Aging

### 7.1. Current Evidence and Source Attribution

The available evidence is insufficient to establish a clinically useful circulating circRNA biomarker for sarcopenia. This is a limitation of study coverage and validation, not evidence that circRNAs lack a biological relationship with muscle aging. Tissue mechanisms, blood associations, and diagnostic usefulness address different questions. Differential expression in a patient cohort would support candidate discovery, but would not by itself establish analytical reproducibility, independent predictive value, or clinical utility. Intended use should be specified before validation, with performance assessed beyond age and standard phenotyping [28].

Human blood evidence extends beyond exercise studies. In 306 InCHIANTI participants, peripheral-blood circFNDC3B expression showed nominal cross-sectional and longitudinal associations with grip strength [81]. These associations did not meet the study’s multiple-testing threshold. This was a cellular blood-RNA analysis, not a cell-free serum or plasma sarcopenia-validation study. It supports further investigation without establishing diagnostic performance.

Fitz et al. reported differential circRNA annotations in total serum extracellular vesicles from older adults stratified by mobility resilience [82]. Sequencing involved 12 participants per group within a 45-person cohort. The circRNA profiles came from total vesicles, whereas the muscle-enriched vesicle arm examined other cargos. Thus, muscle origin cannot be assigned to these circRNA signals. This discovery evidence concerns gait-speed phenotypes, not a validated sarcopenia test.

In 11 marathon runners, plasma circMBOAT2 decreased 24 h after the race [83]. This provides a circulating exercise-response comparator without establishing muscle origin or sarcopenia specificity. Table 6 separates plasma, cellular blood, and serum-vesicle evidence. The reviews by Shin et al. and Li et al. [22,84] provided source-tracing leads and were not counted as independent patient cohorts.

**Table 6:** Human blood circRNA studies relevant to aging or exercise and their limits for sarcopenia biomarker development.

Candidate	Population and Sample	Matrix and Timing	Reported Finding	Unmet Sarcopenia-Biomarker Domains
circMBOAT2 [83]	Marathon runners (*n* = 11); within-person time course	Plasma; baseline, immediately after the race, and 24 h after the race	Lower abundance at 24 h; correlations with exercise-related measures	No older or clinically defined sarcopenia cohort; no skeletal-muscle source attribution; no independent validation, diagnostic or prognostic performance, or decision utility
circFNDC3B [81]	InCHIANTI (*n* = 306)	Cellular peripheral-blood RNA; repeated follow-up	Nominal grip-strength associations	Not cell-free serum/plasma; no independent sarcopenia diagnostic validation
circRNA annotations [82]	Health ABC (45 total; sequencing 12 per group)	Total serum extracellular vesicles; gait-speed groups	Differential RNA profiles	Mobility discovery, not sarcopenia validation; circRNA muscle source unresolved

**Note:** Cellular peripheral-blood RNA, cell-free plasma, and serum extracellular vesicles are different specimen classes. The table summarizes discovery and association evidence, not validated diagnosis of clinically defined sarcopenia. Gait-speed grouping alone is not a sarcopenia diagnosis. Further evaluation should establish junction-level analytical identity, independent validation, and added clinical value. Unmet domains correspond to Table 7.

**Table 7:** Proposed readiness criteria for circRNA biomarkers of skeletal muscle aging and sarcopenia. B1–B8 correspond to the sequential biomarker checkpoints in Figure 3. General evidence for extracellular RNA and platelet circRNA is summarized in refs. [68,69,70].

Domain	Minimum Requirement	Why It Matters
B1 Analytical identity	Back-splice junction sequence; assay specificity; discrimination from host/linear RNA; reproducibility	Avoids measuring the wrong RNA species
B2 Pre-analytical robustness	Tube type; processing delay; freeze–thaw; hemolysis; platelet/cell contamination; storage	Essential for plasma, serum, or extracellular-vesicle studies
B3 Source attribution	Muscle enrichment or orthogonal evidence of muscle contribution; cell-type localization where possible	Prevents generic circulating RNA from being mislabeled as muscle-specific
B4 Sarcopenia phenotype	Accepted sarcopenia criteria; strength; muscle quantity/quality; physical performance	Links molecular signal to clinical phenotype
B5 Independent validation	Separate discovery and validation cohorts; external laboratory where feasible	Limits overfitting and batch-specific findings
B6 Longitudinal prediction	Prediction of decline beyond age and standard variables	Demonstrates incremental utility
B7 Intervention responsiveness	Prespecified response to exercise/nutrition/therapy with appropriate timing	Supports pharmacodynamic use
B8 Clinical utility	Effect size, calibration, discrimination and clinically relevant thresholds for intended use	Separates association from clinically useful classification

**Figure 3:**
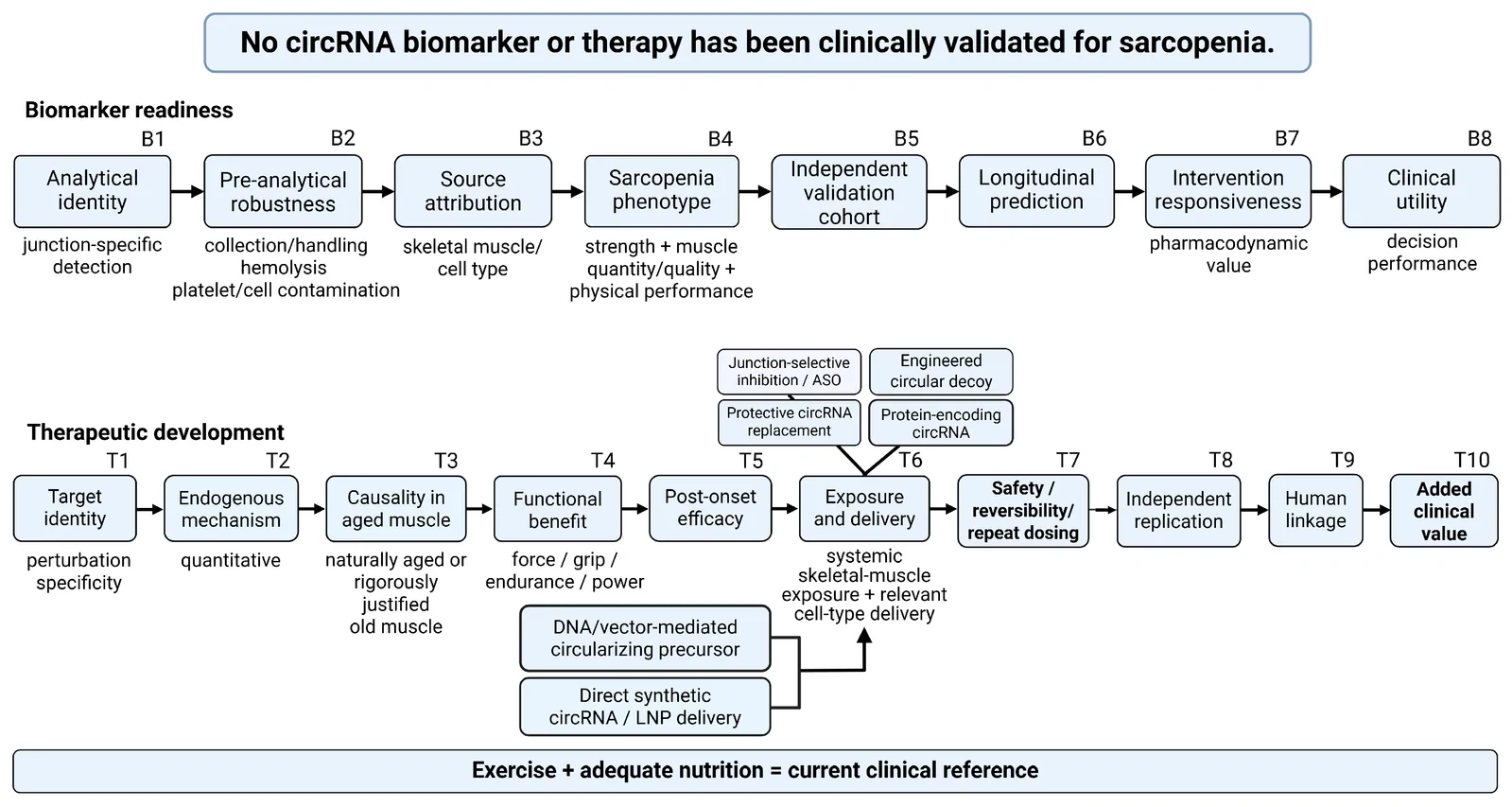
Sequential development checkpoints for circRNA biomarkers and therapeutics in skeletal muscle aging. B1–B8 denote analytical identity, pre-analytical robustness, source attribution, sarcopenia phenotype, independent validation, longitudinal prediction, intervention responsiveness, and clinical utility. T1–T10 denote target identity, endogenous mechanism, causality in aged muscle, functional benefit, post-onset efficacy, exposure and delivery, safety, reversibility and repeat dosing, independent replication, human linkage, and added clinical value. The alphanumeric identifiers mark checkpoint order and are not ratings of current achievement. Arrows show the intended development sequence only; they do not imply biological causality or the likelihood of regulatory approval. The upper statement summarizes the targeted literature reviewed here: no circRNA biomarker or therapy is clinically validated for sarcopenia. Illustrative therapeutic modalities include junction-selective antisense oligonucleotides (ASOs), circular decoys, protective circRNA replacement, protein-encoding circRNAs, vector-mediated expression, and direct synthetic circRNA or lipid nanoparticle (LNP) delivery. Detailed criteria and decision rules are provided in Table 7 and Table 8. Exercise and adequate nutrition provide the current clinical reference against which added therapeutic value should be tested. ASO, antisense oligonucleotide; circRNA, circular RNA; LNP, lipid nanoparticle. Created in BioRender. Lee, W. (2026) https://BioRender.com/f3eln06, accessed on 12 September 2026.

**Table 8:** Criteria for continued development of circRNA therapeutics for skeletal muscle aging. T1–T10 correspond to the sequential therapeutic checkpoints in Figure 3.

Criterion	Question	Decision Rule
T1 Target identity	Is the circular species unambiguously distinguished from host transcript and linear isoforms?	Stop if identity or perturbation specificity is unresolved.
T2 Endogenous mechanism	Does the proposed interaction operate at endogenous abundance with direct evidence and rescue/epistasis?	Revise if the mechanism depends only on prediction or supraphysiological overexpression.
T3 Causality in aged muscle	Does junction-selective manipulation alter a relevant phenotype in naturally aged or rigorously justified old muscle?	Comparator atrophy alone is insufficient.
T4 Functional benefit	Does intervention improve force, grip, endurance, power or another meaningful functional endpoint?	Mass or cross-sectional area alone does not meet this criterion.
T5 Post-onset efficacy	Can benefit occur after the phenotype is established?	Prioritize post-onset evidence over prophylaxis alone.
T6 Exposure and delivery	Can the modality achieve reproducible exposure across relevant muscles and cell types?	Local injection is not systemic delivery.
T7 Safety, reversibility, and repeat dosing	Are innate immunity, host-gene effects, off-targets, encoded products, and repeat dosing assessed?	Use aged and multimorbid models when feasible.
T8 Independent replication	Is the core result reproduced with independent reagents or cohorts and preferably another laboratory?	Concentration in one investigator network lowers readiness.
T9 Human linkage	Is the target associated with human muscle aging, strength or clinically defined sarcopenia?	Sequence conservation alone is insufficient.
T10 Added clinical value	Does the strategy add to exercise and nutrition or address patients inadequately served by them?	Defines a credible development path.

### 7.2. Minimum Biomarker Validation Pathway

Four use cases require separate validation. A tissue marker may reflect biological muscle age or pathway state. A circulating diagnostic marker may associate with clinically defined sarcopenia. A prognostic marker may predict future functional decline, while a pharmacodynamic marker may change with exercise, nutrition, or molecular intervention. Success in one role does not establish performance in another.

Biopsy provides direct source attribution. Age-associated changes in circSnd1 and circDdb1, together with aging/exercise profiling datasets, show that circRNA expression can reflect an aged-muscle state [63,64,71]. Screening utility is nevertheless limited, and biopsy measurements are sensitive to muscle group, fiber composition, physical activity, recent exercise, sex, comorbidity, and cellular admixture. A credible tissue marker requires an independent cohort, a junction-specific assay, and an association with strength, muscle quality, or longitudinal decline, independent of age alone.

Repeated blood sampling makes circulating circRNAs attractive, but the same accessibility creates source ambiguity. Hemolysis can distort extracellular RNA measurements, while the high abundance of circRNAs in platelets and their selective release through platelet-derived extracellular vesicles create a circRNA-specific source problem [68,69,70]. Tube choice, plasma preparation, processing delay, and storage therefore require prospective validation. Exercise, inflammation, renal function, and tissue injury add biological variability. A plasma circRNA can be reproducible yet unrelated to skeletal muscle.

A circulating candidate should be considered muscle-derived only when muscle contribution is supported by tissue enrichment, paired tissue-blood behavior, cell-type localization, release experiments, or another orthogonal strategy. The clinical association should also survive relevant confounders. Evidence from independent human cohorts with prespecified sarcopenia criteria, diagnostic-performance metrics, longitudinal follow-up, and external validation remains sparse. The proposed biomarker-validation pathway is summarized in Table 7.

## 8. Therapeutic Intervention Concepts for Aging Muscle

### 8.1. Clinical Comparators and Endogenous Targets

Exercise, particularly resistance-oriented training, has the strongest intervention evidence for sarcopenia and improves strength and physical performance across many trials [85]. Nutritional support can be useful, especially when intake is inadequate, but meta-analyses differ on the extent to which protein supplementation enhances resistance training outcomes across populations [86,87]. A circRNA therapy should ultimately be assessed as an adjunct to established care or as an option for people unable to obtain sufficient benefit from exercise and nutrition.

Exercise also provides a mechanistically informative perturbation for RNA biology. In naturally aging rats, distinct exercise regimens alter microRNA profiles together with atrophy-related phenotypes [88], and aging circRNA profiles are exercise-responsive [71]. Human studies should test whether circRNAs track or mediate adaptation to resistance exercise, with longitudinal sampling alongside resting old–young comparisons.

Exercise-responsive circRNAs can also guide exploratory studies of compounds investigated for aging-related muscle dysfunction. CircBBS9 is a candidate because its overexpression changed atrophy- and mitochondrial-program transcripts in myotubes [71]. It could serve as a prespecified mechanistic readout in studies of compounds such as resveratrol. This is a proposed experiment, not evidence that resveratrol acts through circBBS9. CircFUT10 could similarly be tested in aged muscle stem cells, while circSnd1 and circDdb1 offer mechanistically defined targets for testing effects on protein stability or translation [27,63,64].

A compound-induced change in circRNA abundance should initially be interpreted as a pharmacodynamic association. To establish mediation, junction-selective depletion or expression rescue should alter the compound’s effect while preserving the linear host transcript. Target engagement, autophagic or mitochondrial flux where relevant, and muscle function should be measured separately. A compound–circRNA interaction should advance only if the benefit depends on the candidate and persists in an aged model without unacceptable toxicity. The studies summarized here do not establish these circRNAs as validated mediators of an anti-aging compound.

For pathogenic candidates such as circSnd1 or circDdb1, junction-selective inhibition is the most direct concept. Antisense oligonucleotides (ASOs) or related RNA-targeting strategies could in principle reduce the circle while sparing the host transcript, but selectivity must be demonstrated experimentally. In older adults, repeat dosing, renal and hepatic handling, innate immune activation, off-target hybridization, and distribution across a large muscle mass become central development questions. Dose–exposure–response relationships in relevant muscles are more informative than local proof-of-concept injection alone.

### 8.2. Protective and Engineered circRNA Modalities

Protective circRNAs could be augmented by direct RNA delivery or by DNA vectors that express a circularizing precursor. These are pharmacologically different modalities. Direct circRNA administration provides a defined RNA dose but faces constraints on uptake, endosomal escape, and biodistribution. Vector-mediated expression may provide longer exposure but is less reversible and introduces vector immunity and redosing issues. These distinctions preclude the blanket statement that an adeno-associated virus (AAV) ‘delivers circRNA.’

Engineered circmiR-29b shows that circular topology can serve as a therapeutic platform, not only as an endogenous target [73]. Engineered circular RNAs can also support prolonged protein expression [18,89]. In some settings, a designed molecule may be more tractable than manipulating a low-abundance endogenous circRNA, as sequence, potency, translation, purity, and manufacturing can be deliberately optimized.

Broader circRNA technologies have entered early human testing beyond skeletal muscle. RXRG001 is a lipid-nanoparticle (LNP)-formulated, protein-encoding circRNA registered for radiation-induced xerostomia. RXIM002 is a circRNA-LNP product designed for in vivo CD19-directed chimeric antigen receptor (CAR) T-cell engineering [90,91]. These registrations establish entry into early clinical testing, but they do not establish skeletal-muscle targeting, sarcopenia efficacy, or long-term safety in older adults. A targeted ClinicalTrials.gov search updated on 12 September 2026 did not identify an interventional circRNA trial for sarcopenia or skeletal-muscle wasting. The registered circRNA-encoded peptide dendritic-cell vaccine in breast cancer [92] is an ex vivo cellular vaccine and does not provide evidence for direct systemic circRNA delivery.

### 8.3. Delivery Constraints for Systemic Muscle Treatment

Skeletal muscle is a large, distributed organ. Effective systemic therapy may require exposure across multiple limb and respiratory muscles while limiting exposure to the liver, spleen, heart, and immune system. LNPs can encapsulate in vitro-transcribed circRNA and provide a clinically familiar manufacturing concept [20,93], but generic LNPs are not inherently muscle-specific. Local intramuscular success should not be interpreted as evidence that a modality can treat generalized sarcopenia.

The required target cell may also differ depending on the mechanism. A myofiber-directed therapy may not correct satellite-cell, fibro-adipogenic progenitor, motor-neuron, vascular, or immune components of aging. The delivery strategy should follow causal cell localization and not precede it.

## 9. Translational Evaluation Framework

### 9.1. Criteria for Continued Development

Table 8 and the T1–T10 therapeutic track in Figure 3 define sequential criteria for deciding whether a candidate should advance, be redesigned, or be discontinued. The identifiers indicate checkpoint order rather than current achievement. Failure at an early criterion should not be offset by additional descriptive data.

### 9.2. Replication and Model Relevance

Several of the strongest muscle-wasting circRNA studies are technically rigorous and provide substantial mechanistic or functional evidence. Multiple advanced candidates were also developed within overlapping investigator networks and related experimental platforms. This concentration does not invalidate the findings, but it limits the current evidence for generalizability. circTmeff1, circDdb1, circSnd1, and engineered circmiR-29b share investigators across studies [63,64,65,73]. Independent reproduction of direction, mechanism, and function should precede expensive delivery, toxicology, or manufacturing programs.

Older organisms differ in immune responsiveness, renal and hepatic handling, tissue perfusion, matrix stiffness, regenerative capacity, neuromuscular reserve, and multimorbidity. A dose that changes a circRNA in young denervated muscle may have different exposure, safety, and efficacy in aged tissue. Translational experiments should include naturally aged animals or a rigorously justified aging model and should report sex, activity, frailty-related phenotype, and baseline function. Across the candidate literature, sex and baseline activity were not reported consistently enough for reliable cross-study stratification; this reporting gap limits generalizability.

## 10. Emerging Directions

### 10.1. Mechanotransduction and Cell-Resolved Discovery

Mechanotransduction is an attractive but underdeveloped bridge between circRNAs and aging muscle. Nuclear mechanobiology and YAP/TAZ signaling change with aging and unloading [48,49,50,51], while work on the TPM1 super-enhancer and circTPM1 links a skeletal-muscle circRNA program to mechanotransduction and regeneration [80]. The next experiments should identify load-responsive circRNAs in old human muscle, localize them to the relevant cell type, quantify endogenous abundance, and test junction-specific causality under controlled mechanical conditions. They should then determine whether the candidates modify YAP/TAZ, myocardin-related transcription factor/serum response factor (MRTF/SRF), focal-adhesion, ion-channel, or mitochondrial responses. Recent discussion of circRNA-RBP mechanisms in exercise remodeling also supports moving beyond default sponge diagrams [26].

Whole-muscle RNA averages cell populations that change with age. Long-read sequencing can improve isoform definition, while spatial and single-cell-compatible approaches can identify where a circRNA and its molecular partners coexist. These methods are particularly important for low-abundance ceRNA claims and for blood-biomarker source attribution. Standard poly(A)-based single-cell workflows do not uniformly capture circRNAs. The method choice should match the circular species being tested.

### 10.2. Integrating Biomarkers, Interventions, and Mechanism

A strong translational program should connect biomarker and therapeutic development. A tissue or circulating circRNA that reflects target engagement could support dose selection, stratification, and proof of mechanism. An intervention-responsive circRNA may also be useful as a pharmacodynamic marker even if it is not causal. Separating causal targets from useful markers prevents the assumption that every biomarker should also be a therapeutic target.

Muscle circRNA research should increasingly test RBP interactions, translation, localization-dependent functions, and host-gene relationships instead of defaulting to a circRNA-miRNA-mRNA diagram [11,26]. The strongest current wasting examples already support this shift. circSnd1 acts through protein stability, circDdb1 and circTmeff1 include protein-coding mechanisms, and engineered circles operate as designed therapeutic molecules. Mechanistic diversity is central to identifying biology that can withstand translational scrutiny.

## 11. Conclusions

CircRNA biology now intersects meaningfully with skeletal-muscle aging, although the maturity of individual claims differs sharply. The most convincing studies combine unambiguous circular identity, specific perturbation, a quantitatively credible mechanism, relevant in vivo causality, and functional endpoints. circSnd1 and circDdb1 currently provide the strongest functional preclinical evidence and are associated with aged human skeletal muscle, but neither has clinical validation. circFUT10, circBBS9, and circNEB address regenerative, discovery, and histological aspects of aging and require deeper functional testing. circGLIS3 is relevant to muscle quality but should be examined directly in aging before it is considered a sarcopenia mechanism.

Emerging blood and serum-vesicle associations support further investigation but do not establish a clinical sarcopenia test. Progress in biomarker development requires junction-level analytical validation, pre-analytical robustness, source attribution, accepted sarcopenia phenotyping, independent cohorts, and longitudinal or intervention-linked performance. Therapeutic development depends on systemic muscle exposure, post-onset functional efficacy in aged organisms, safety, reversibility, and independent replication. CircRNA platforms have entered human clinical development outside skeletal muscle, but this does not establish skeletal-muscle delivery, efficacy, or long-term safety. Future work should focus on a small number of candidates tested rigorously against these requirements.

## Figures and Tables

**Table 1:** Sources and search concepts used in the critical narrative review.

Source or Purpose	Terms and Scope
Core PubMed concepts	‘circular RNA’ or ‘circRNA’, combined with skeletal muscle aging/ageing, sarcopenia, muscle atrophy, or named circRNAs
Models and interventions	Denervation, disuse, immobilization, dexamethasone, cachexia, exercise, satellite cell, and myosteatosis
Mechanisms and translation	Mitochondria, inflammation, mechanotransduction, Yes-associated protein (YAP), biomarker, and delivery
ClinicalTrials.gov	‘circRNA’ or ‘circular RNA’ with sarcopenia, skeletal muscle wasting, or muscle atrophy, plus named platforms and trial identifiers. Original search through 12 September 2026
Revision biomarker searches	(circRNA OR circular RNA) AND sarcopenia AND (serum OR plasma OR circulating); expanded with muscle aging/ageing and blood. PubMed, 27 September 2026
Revision source tracing	Reviewer-suggested PubMed identifiers (PMIDs) 38264571 and 41615487, their accessible references, and targeted searches for circRNA senescence, endocrine secretion, and RNA-binding protein (RBP) interactions
Interpretation	Narrative, English-language, targeted coverage. Cellular blood RNA, cell-free plasma/serum, tissue measurements, and clinical validation were evaluated separately

**Table 2:** Retraction audit of circRNA reports excluded from the positive evidence synthesis.

Retracted Report	Retraction Record	Risk of Confusion	Treatment in This Review
Exosomal ciRS-133 report in gastric cancer	Publisher retraction verified (2023)	The report linked an extracellular circRNA to systemic adipose browning and could be carried into metabolic or muscle narratives.	Excluded from the positive evidence synthesis.
circ-FoxO3 report on myoblast differentiation	Publisher retraction verified (2020)	The original claim concerned a direct myoblast differentiation phenotype.	Excluded from mechanistic and developmental evidence.
2018 bovine circFUT10 and miR-133a report	Publisher retraction verified (2026)	The circRNA name can be confused with the separate 2021 study in aged skeletal-muscle stem cells [27].	The 2018 report is excluded. The 2021 study is evaluated separately.

**Table 3:** Evidence levels used to classify circRNA studies in skeletal muscle aging and related wasting contexts.

Evidence Level	Minimum Evidence	Interpretation
Level 1 Discovery and association	Differential expression, profiling, correlation, network prediction or exercise responsiveness	Hypothesis generation; no causal inference
Level 2 Mechanistic	Circular identity + candidate perturbation + directly tested interaction or pathway	Mechanism supported; in vivo aging or wasting relevance incomplete
Level 3 In vivo causal	Manipulation changes an aging- or wasting-relevant tissue phenotype in vivo	Causality supported; therapeutic meaning depends on timing and endpoint
Level 4 Functional intervention	In vivo intervention with a meaningful functional endpoint and concordant tissue evidence	Highest preclinical functional class. Post-onset efficacy is reported separately as a modifier.

Human linkage, species, treatment timing, locus of action, and engineered or endogenous status are summarized separately in Supplementary Table S1 and do not change the evidence level by themselves.

**Table 4:** Selected circRNA studies with direct aging linkage through aging-related models or aged-human-muscle association. Human linkage is assessed separately and does not imply human efficacy.

Candidate and Level	Aging Context and Models	Mechanistic Evidence	Strongest Endpoint	Human Linkage	Principal Limitation
circSnd1 Level 4	Aged human muscle (*n* = 5 per age group), human myotubes, mice, and C2C12 cells; multiple catabolic stressors	FAT10-EEF1A1 interaction and EEF1A1 stabilization	Muscle mass or CSA, grip, and contractile endpoints	Aged-muscle association and human myotubes	No clinically adjudicated sarcopenia, longitudinal outcome, or independent validation
circDdb1 Level 4	Aged human muscle (five young and five aged samples), mice, and C2C12 cells; multiple atrophy conditions	Encodes circDdb1-867aa; eEF2 Thr56 phosphorylation and translation control	Muscle mass or CSA, grip, endurance, and contractile endpoints	Aged-muscle association	No accepted sarcopenia phenotype, longitudinal outcome, or independent validation
circFUT10 Level 2	Aged skeletal-muscle stem cells	miR-365a-3p-HOXA9 axis	Cellular regenerative phenotypes	None reported	No aged-organism mass or functional rescue
circBBS9 Level 1 with preliminary cellular support	Aged mouse muscle and differentiated C2C12 myotubes	Exercise responsiveness and overexpression-linked mRNA changes	Transcript readouts; no candidate-directed aged-animal functional test	None	Interaction-specific mechanism and in vivo efficacy remain unresolved
circNEB Level 3	Four 40-month-old rabbits with paired local hindlimb injections; bovine myoblasts; mouse and tree shrew injury models	Translated peptide; SKP1/p27-Kip1- and TPM1-related mechanisms	Short-term aged-rabbit histology	None	*n* = 4 aged rabbits; paired-limb local plasmid design; blinding and statistical unit not clearly reported; short-term histology; no functional endpoint

C2C12, murine myoblast cell line; CSA, cross-sectional area; eEF2, eukaryotic elongation factor 2; EEF1A1, eukaryotic translation elongation factor 1 alpha 1; FAT10, ubiquitin D; HOXA9, homeobox A9; SKP1, S-phase kinase-associated protein 1; TPM1, tropomyosin 1.

**Table 5:** Non-aging atrophy and aging-adjacent studies used to test mechanistic portability without substituting for direct sarcopenia evidence.

Candidate	Comparator Context	Mechanistic Axis	Strongest Endpoint	Level	Use and Limitation in an Aging Review
circTmeff1	Denervation; immobilization; inflammatory wasting	TDP-43; mitochondrial stress and cGAS-STING; encoded protein	Functional rescue, including established atrophy	4	High mechanistic portability; aging/human sarcopenia not directly tested
Engineered circmiR-29b	Immobilization and other atrophy models	Synthetic miR-29b decoy	Post-onset attenuation of established immobilization atrophy without a reported functional endpoint	3	Engineered modality proof. No direct aging model or functional endpoint.
circANAPC7	Pancreatic-cancer cachexia	Tumor PHLPP2-AKT-TGF-beta axis	Muscle mass, fiber CSA, and grip strength	4	Systemic/tumor-mediated; not muscle-intrinsic aging biology
circGLIS3	HFD-associated muscle fat infiltration	Suppression of intramuscular adipogenesis	Reduced fat infiltration in vivo	3	Aging-adjacent muscle-quality evidence; not an aging model
circAGO3	Inflammatory atrophy	miR-34b-5p-TRAF3-NF-kappaB	Causal in vivo atrophy phenotype	3	Chicken; species-limited
circCCDC91	Dexamethasone-related atrophy/myogenesis	miR-15 family-IRS1-IGF1-PI3K-AKT	Cell-level rescue	2	Primarily in vitro and chicken
circSMOX/denervation profiles	Dexamethasone, ALS-related, or denervation-associated expression	Not fully causally resolved	Association/profiling	1	Hypothesis pool only
circAtxn10	Human myogenic-cell discovery; C2C12 differentiation	Proposed miR-143-3p–CHRNA1 axis	Myogenic cellular phenotype	2	Human-cell discovery with murine mechanistic validation; no direct aging study
circTPM1	Myogenic differentiation and regeneration	MYH10 binding and MYL3-dependent actomyosin assembly downstream of TPM1_SE	Bovine cellular mechanism with cross-species super-enhancer evidence	2	Mechanotransduction relevance. No chronological aging, sarcopenia, or circRNA-specific in vivo test.

AKT, protein kinase B; ALS, amyotrophic lateral sclerosis; cGAS-STING, cyclic GMP-AMP synthase-stimulator of interferon genes; CHRNA1, cholinergic receptor nicotinic alpha 1 subunit; CSA, cross-sectional area; HFD, high-fat diet; IGF1, insulin-like growth factor 1; IRS1, insulin receptor substrate 1; MYH10, myosin heavy chain 10; MYL3, myosin light chain 3; NF-kappaB, nuclear factor kappa B; PHLPP2, PH domain leucine-rich repeat protein phosphatase 2; PI3K, phosphoinositide 3-kinase; TDP-43, transactive response DNA-binding protein 43; TGF-beta, transforming growth factor beta; TPM1_SE, TPM1 super-enhancer; TRAF3, TNF receptor-associated factor 3.

## Data Availability

No new data were created or analyzed in this study. Data sharing is not applicable to this article. All information evaluated in this review is available in the cited publications and public clinical trial registries.

## Supplementary Materials

The following supporting information can be downloaded at: https://www.mdpi.com/article/10.3390/ijms27198844/s1. Table S1: Molecular identity and modifiers of studies in Table 4 and Table 5. Cited studies [27,58,60,63,64,65,71,72,73,75,76,77,78,79,80] also appear in the main reference list.

## References

[1] Cruz-Jentoft A.J., Bahat G., Bauer J., Boirie Y., Bruyère O., Cederholm T., Cooper C., Landi F., Rolland Y., Sayer A.A. (2019). Sarcopenia: Revised European consensus on definition and diagnosis. Age Ageing.

[2] Chen L.K., Woo J., Assantachai P., Auyeung T.W., Chou M.Y., Iijima K., Jang H.C., Kang L., Kim M., Kim S. (2020). Asian Working Group for Sarcopenia: 2019 Consensus Update on Sarcopenia Diagnosis and Treatment. J. Am. Med. Dir. Assoc..

[3] Kirk B., Cawthon P.M., Arai H., Ávila-Funes J.A., Barazzoni R., Bhasin S., Binder E.F., Bruyere O., Cederholm T., Chen L.K. (2024). The Conceptual Definition of Sarcopenia: Delphi Consensus from the Global Leadership Initiative in Sarcopenia (GLIS). Age Ageing.

[4] Larsson L., Degens H., Li M., Salviati L., Lee Y.I., Thompson W., Kirkland J.L., Sandri M. (2019). Sarcopenia: Aging-Related Loss of Muscle Mass and Function. Physiol. Rev..

[5] Grima-Terrén M., Campanario S., Ramírez-Pardo I., Cisneros A., Hong X., Perdiguero E., Serrano A.L., Isern J., Muñoz-Cánoves P. (2024). Muscle aging and sarcopenia: The pathology, etiology, and most promising therapeutic targets. Mol. Asp. Med..

[6] Lopez-Otin C., Blasco M.A., Partridge L., Serrano M., Kroemer G. (2023). Hallmarks of aging: An expanding universe. Cell.

[7] Ferri E., Marzetti E., Calvani R., Picca A., Cesari M., Arosio B. (2020). Role of Age-Related Mitochondrial Dysfunction in Sarcopenia. Int. J. Mol. Sci..

[8] Chen M., Wang Y., Deng S., Lian Z., Yu K. (2022). Skeletal muscle oxidative stress and inflammation in aging: Focus on antioxidant and anti-inflammatory therapy. Front. Cell Dev. Biol..

[9] Memczak S., Jens M., Elefsinioti A., Torti F., Krueger J., Rybak A., Maier L., Mackowiak S.D., Gregersen L.H., Munschauer M. (2013). Circular RNAs are a large class of animal RNAs with regulatory potency. Nature.

[10] Kristensen L.S., Andersen M.S., Stagsted L.V.W., Ebbesen K.K.E., Hansen T.B., Kjems J. (2019). The biogenesis, biology and characterization of circular RNAs. Nat. Rev. Genet..

[11] Liu C.X., Chen L.L. (2022). Circular RNAs: Characterization, cellular roles, and applications. Cell.

[12] Jeck W.R., Sorrentino J.A., Wang K., Slevin M.K., Burd C.E., Liu J., Marzluff W.F., Sharpless N.E. (2013). Circular RNAs are abundant, conserved, and associated with ALU repeats. RNA.

[13] Salzman J., Gawad C., Wang P.L., Lacayo N., Brown P.O. (2012). Circular RNAs are the predominant transcript isoform from hundreds of human genes in diverse cell types. PLoS ONE.

[14] Ashwal-Fluss R., Meyer M., Pamudurti N.R., Ivanov A., Bartok O., Hanan M., Evantal N., Memczak S., Rajewsky N., Kadener S. (2014). circRNA biogenesis competes with pre-mRNA splicing. Mol. Cell.

[15] Hansen T.B., Jensen T.I., Clausen B.H., Bramsen J.B., Finsen B., Damgaard C.K., Kjems J. (2013). Natural RNA circles function as efficient microRNA sponges. Nature.

[16] Legnini I., Di Timoteo G., Rossi F., Morlando M., Briganti F., Sthandier O., Fatica A., Santini T., Andronache A., Wade M. (2017). Circ-ZNF609 Is a Circular RNA that Can Be Translated and Functions in Myogenesis. Mol. Cell.

[17] Yang Y., Fan X., Mao M., Song X., Wu P., Zhang Y., Jin Y., Yang Y., Chen L.L., Wang Y. (2017). Extensive translation of circular RNAs driven by N6-methyladenosine. Cell Res..

[18] Wesselhoeft R.A., Kowalski P.S., Anderson D.G. (2018). Engineering circular RNA for potent and stable translation in eukaryotic cells. Nat. Commun..

[19] O’Leary E., Jiang Y., Kristensen L.S., Hansen T.B., Kjems J. (2025). The therapeutic potential of circular RNAs. Nat. Rev. Genet..

[20] Cao X., Cai Z., Zhang J., Zhao F. (2025). Engineering circular RNA medicines. Nat. Rev. Bioeng..

[21] Knupp D., Miura P. (2018). CircRNA accumulation: A new hallmark of aging?. Mech. Ageing Dev..

[22] Shin Y.J., Kwon K.S., Suh Y., Lee K.P. (2024). The role of noncoding RNAs in muscle aging: Regulatory mechanisms and therapeutic potential. Front. Mol. Biosci..

[23] Yu P., Qi Y., Huang H., Wang H., Xu J. (2025). CircularRNA and Musculoskeletal Diseases. Adv. Exp. Med. Biol..

[24] Huang C.J., Choo K.B. (2025). Circular RNAs and host genes act synergistically in regulating cellular processes and functions in skeletal myogenesis. Gene.

[25] Greco S., Cardinali B., Falcone G., Martelli F. (2018). Circular RNAs in muscle function and disease. Int. J. Mol. Sci..

[26] Liu J., Yang Y., Chen H., Liu M., Mao Z., Zheng M. (2026). Regulatory roles of the circRNA-RBP axis in exercise-induced skeletal muscle remodeling: Mechanistic controversies and translational illusions. Front. Genet..

[27] Zhu M., Lian C., Chen G., Zou P., Qin B.G. (2021). CircRNA FUT10 regulates the regenerative potential of aged skeletal muscle stem cells by targeting HOXA9. Aging.

[28] Huang N., Ge M., Liu X., Tian X., Yin P., Bao Z., Cao F., Shyh-Chang N., Dong B., Dai L. (2024). A framework of biomarkers for skeletal muscle aging: A consensus statement by the Aging Biomarker Consortium. Life Med..

[29] Hughes D.C., Baehr L.M., Waddell D.S., Bodine S.C. (2022). Ubiquitin Ligases in Longevity and Aging Skeletal Muscle. Int. J. Mol. Sci..

[30] Fujita S., Glynn E.L., Timmerman K.L., Rasmussen B.B., Volpi E. (2009). Supraphysiological hyperinsulinaemia is necessary to stimulate skeletal muscle protein anabolism in older adults: Evidence of a true age-related insulin resistance of muscle protein metabolism. Diabetologia.

[31] Durham W.J., Casperson S.L., Dillon E.L., Keske M.A., Paddon-Jones D., Sanford A.P., Hickner R.C., Grady J.J., Sheffield-Moore M. (2010). Age-related anabolic resistance after endurance-type exercise in healthy humans. FASEB J..

[32] Moro T., Brightwell C.R., Deer R.R., Graber T.G., Galvan E., Fry C.S., Volpi E., Rasmussen B.B. (2018). Muscle protein anabolic resistance to essential amino acids does not occur in healthy older adults before or after resistance exercise training. J. Nutr..

[33] Jing Y., Zuo Y., Sun L., Yu Z.R., Ma S., Hu H., Zhao Q., Huang D., Zhang W., Belmonte J.C.I. (2023). SESN1 is a FOXO3 effector that counteracts human skeletal muscle ageing. Cell Prolif..

[34] Zhang J., Hu M., Wu X., Guo M., Ma Y., Qiu J., Wang S., Cao Y., Zhong Y., Chen F. (2026). Skeletal Muscle HSF1 Alleviates Age-Associated Sarcopenia and Mitochondrial Function Decline via SIRT3-PGC1alpha Axis. Adv. Sci..

[35] Affourtit C., Carre J.E. (2024). Mitochondrial involvement in sarcopenia. Acta Physiol..

[36] Grevendonk L., Connell N.J., McCrum C., Fealy C.E., Bilet L., Bruls Y.M.H., Mevenkamp J., Schrauwen-Hinderling V.B., Jörgensen J.A., Moonen-Kornips E. (2021). Impact of aging and exercise on skeletal muscle mitochondrial capacity, energy metabolism, and physical function. Nat. Commun..

[37] Scudese E., Marshall A.G., Vue Z., Exil V., Rodriguez B.I., Demirci M., Vang L., López E.G., Neikirk K., Shao B. (2025). 3D Mitochondrial Structure in Aging Human Skeletal Muscle: Insights Into MFN-2-Mediated Changes. Aging Cell.

[38] Sousa-Victor P., Gutarra S., García-Prat L., Rodriguez-Ubreva J., Ortet L., Ruiz-Bonilla V., Jardí M., Ballestar E., González S., Serrano A.L. (2014). Geriatric muscle stem cells switch reversible quiescence into senescence. Nature.

[39] Cosgrove B.D., Gilbert P.M., Porpiglia E., Mourkioti F., Lee S.P., Corbel S.Y., Llewellyn M.E., Delp S.L., Blau H.M. (2014). Rejuvenation of the muscle stem cell population restores strength to injured aged muscles. Nat. Med..

[40] Fry C.S., Lee J.D., Mula J., Kirby T.J., Jackson J.R., Liu F., Yang L., Mendias C.L., Dupont-Versteegden E.E., McCarthy J.J. (2015). Inducible depletion of satellite cells in adult, sedentary mice impairs muscle regenerative capacity without affecting sarcopenia. Nat. Med..

[41] Panda A.C., Grammatikakis I., Kim K.M., De S., Martindale J.L., Munk R., Yang X., Abdelmohsen K., Gorospe M. (2017). Identification of senescence-associated circular RNAs (SAC-RNAs) reveals senescence suppressor CircPVT1. Nucleic Acids Res..

[42] Dowling P., Gargan S., Swandulla D., Ohlendieck K. (2023). Fiber-Type Shifting in Sarcopenia of Old Age: Proteomic Profiling of the Contractile Apparatus of Skeletal Muscles. Int. J. Mol. Sci..

[43] Murgia M., Toniolo L., Nagaraj N., Ciciliot S., Vindigni V., Schiaffino S., Reggiani C., Mann M. (2017). Single Muscle Fiber Proteomics Reveals Fiber-Type-Specific Features of Human Muscle Aging. Cell Rep..

[44] Hepple R.T., Rice C.L. (2016). Innervation and neuromuscular control in ageing skeletal muscle. J. Physiol..

[45] Soendenbroe C., Heisterberg M.F., Schjerling P., Karlsen A., Kjaer M., Andersen J.L., Mackey A.L. (2019). Molecular indicators of denervation in aging human skeletal muscle. Muscle Nerve.

[46] Sarto F., Franchi M.V., McPhee J.S., Stashuk D.W., Paganini M., Monti E., Rossi M., Sirago G., Zampieri S., Motanova E.S. (2024). Neuromuscular impairment at different stages of human sarcopenia. J. Cachexia Sarcopenia Muscle.

[47] Lukasiewicz C.J., Tranah G.J., Evans D.S., Coen P.M., Barnes H.N., Huo Z., Esser K.A., Zhang X., Wolff C., Wu K. (2024). Higher expression of denervation-responsive genes is negatively associated with muscle volume and performance traits in the Study of Muscle, Mobility, and Aging (SOMMA). Aging Cell.

[48] Iyer S.R., Folker E.S., Lovering R.M. (2021). The Nucleoskeleton: Crossroad of Mechanotransduction in Skeletal Muscle. Front. Physiol..

[49] van Ingen M.J.A., Kirby T.J. (2021). LINCing Nuclear Mechanobiology with Skeletal Muscle Mass and Function. Front. Cell Dev. Biol..

[50] Iyer S.R., Hsia R.C., Folker E.S., Lovering R.M. (2021). Age-dependent changes in nuclear-cytoplasmic signaling in skeletal muscle. Exp. Gerontol..

[51] Kwon Y. (2025). YAP/TAZ as Molecular Targets in Skeletal Muscle Atrophy and Osteoporosis. Aging Dis..

[52] Schiaffino S., Reggiani C., Akimoto T. (2021). Molecular Mechanisms of Skeletal Muscle Hypertrophy. J. Neuromuscul. Dis..

[53] Lu Y., Bai Y., Li W., Zhou Z., Lai H., Hu X., Yang T., Wang C., Chen Y., Gan K. (2025). Mechanical Stimulation Induces Yap Mediated OCTN2 Transcription to Enhance Carnitine Metabolism in Sarcopenia. J. Cachexia Sarcopenia Muscle.

[54] Watt K.I., Henstridge D.C., Ziemann M., Sim C.B., Montgomery M.K., Samocha-Bonet D., Parker B.L., Dodd G.T., Bond S.T., Salmi T.M. (2021). Yap regulates skeletal muscle fatty acid oxidation and adiposity in metabolic disease. Nat. Commun..

[55] Sladitschek-Martens H.L., Guarnieri A., Brumana G., Zanconato F., Battilana G., Xiccato R.L., Panciera T., Forcato M., Bicciato S., Guzzardo V. (2022). YAP/TAZ activity in stromal cells prevents ageing by controlling cGAS-STING. Nature.

[56] Shen Y., Luo L., Fu H., Xie L., Zhang W., Lu J., Yang M. (2022). Chest computed tomography-derived muscle mass and quality indicators, in-hospital outcomes, and costs in older inpatients. J. Cachexia Sarcopenia Muscle.

[57] Hetherington-Rauth M., Mansfield T.A., Lenchik L., Weaver A.A., Cawthon P.M. (2025). Associations of CT muscle area and density with functional outcomes and mortality across anatomical regions in older men. J. Am. Geriatr. Soc..

[58] Yu S., Wang J., Liu H., Guo J., Kuraz A.B., Pan Y., Li A., Mei C., Cheng G., Zan L. (2025). CircGLIS3 Inhibits Intramuscular Adipogenesis and Alleviates Skeletal Muscle Fat Infiltration. J. Cachexia Sarcopenia Muscle.

[59] Stoll L., Rodríguez-Trejo A., Guay C., Brozzi F., Bayazit M.B., Gattesco S., Menoud V., Sobel J., Marques A.C., Venø M.T. (2020). A circular RNA generated from an intron of the insulin gene controls insulin secretion. Nat. Commun..

[60] Shi X., Yang J., Liu M., Zhang Y., Zhou Z., Luo W., Fung K.M., Xu C., Bronze M.S., Houchen C.W. (2022). Circular RNA ANAPC7 Inhibits Tumor Growth and Muscle Wasting via PHLPP2-AKT-TGF-beta Signaling Axis in Pancreatic Cancer. Gastroenterology.

[61] Denzler R., Agarwal V., Stefano J., Bartel D.P., Stoffel M. (2014). Assessing the ceRNA hypothesis with quantitative measurements of miRNA and target abundance. Mol. Cell.

[62] Ikeda Y., Morikawa S., Nakashima M., Yoshikawa S., Taniguchi K., Sawamura H., Suga N., Tsuji A., Matsuda S. (2023). CircRNAs and RNA-Binding Proteins Involved in the Pathogenesis of Cancers or Central Nervous System Disorders. Noncoding RNA.

[63] Li J., Jin B., Yan Y., Chen Y., Yin X., Ren X., Li Q., Chen J., Wang S., Yang T. (2026). EIF4A3-Induced Circular RNA circSnd1 Promotes Muscle Atrophy and Muscle Ageing by Stabilizing EEF1A1. J. Cachexia Sarcopenia Muscle.

[64] Zhu X., Yang T., Zheng Y., Nie Q., Chen J., Li Q., Ren X., Yin X., Wang S., Yan Y. (2024). EIF4A3-Induced Circular RNA CircDdb1 Promotes Muscle Atrophy through Encoding a Novel Protein CircDdb1-867aa. Adv. Sci..

[65] Chen R., Yang T., Jin B., Xu W., Yan Y., Wood N., Lehmann H.I., Wang S., Zhu X., Yuan W. (2023). CircTmeff1 Promotes Muscle Atrophy by Interacting with TDP-43 and Encoding A Novel TMEFF1-339aa Protein. Adv. Sci..

[66] Pandey P.R., Yang J.H., Tsitsipatis D., Panda A.C., Noh J.H., Kim K.M., Munk R., Nicholson T., Hanniford D., Argibay D. (2020). circSamd4 represses myogenic transcriptional activity of PUR proteins. Nucleic Acids Res..

[67] Chenette D.M., Cadwallader A.B., Antwine T.L., Larkin L.C., Wang J., Olwin B.B., Schneider R.J. (2016). Targeted mRNA Decay by RNA Binding Protein AUF1 Regulates Adult Muscle Stem Cell Fate, Promoting Skeletal Muscle Integrity. Cell Rep..

[68] Kirschner M.B., Edelman J.J., Kao S.C., Vallely M.P., van Zandwijk N., Reid G. (2013). The impact of hemolysis on cell-free microRNA biomarkers. Front. Genet..

[69] Alhasan A.A., Izuogu O.G., Al-Balool H.H., Steyn J.S., Evans A., Colzani M., Ghevaert C., Mountford J.C., Marenah L., Elliott D.J. (2016). Circular RNA enrichment in platelets is a signature of transcriptome degradation. Blood.

[70] Preußer C., Hung L.H., Schneider T., Schreiner S., Hardt M., Moebus A., Santoso S., Bindereif A. (2018). Selective release of circRNAs in platelet-derived extracellular vesicles. J. Extracell. Vesicles.

[71] Guo M., Qiu J., Shen F., Wang S., Yu J., Zuo H., Yao J., Xu S., Hu T., Wang D. (2020). Comprehensive analysis of circular RNA profiles in skeletal muscles of aging mice and after aerobic exercise intervention. Aging.

[72] Huang K., Li Z., Zhong D., Yang Y., Yan X., Feng T., Wang X., Zhang L., Shen X., Chen M. (2024). A Circular RNA Generated from Nebulin (NEB) Gene Splicing Promotes Skeletal Muscle Myogenesis in Cattle as Detected by a Multi-Omics Approach. Adv. Sci..

[73] Li J., Chen R., Zheng Y., Yuan W., Yang T., Zhu X., Yan Y., Jin B., Xu W., Zhang Z. (2022). Engineered Circular RNA CircmiR-29b Attenuates Muscle Atrophy by Sponging MiR-29b. Adv. Ther..

[74] Ding Z., Sun D., Han J., Shen L., Yang F., Sah S., Sui X., Wu G. (2021). Novel noncoding RNA CircPTK2 regulates lipolysis and adipogenesis in cachexia. Mol. Metab..

[75] Zhao X., Tang S., Lei Z., Shen X., Zhang Y., Han S., Yin H., Cui C. (2024). circAGO3 facilitates NF-kappaB pathway-mediated inflammatory atrophy in chicken skeletal muscle via the miR-34b-5p/TRAF3 axis. Int. J. Biol. Macromol..

[76] Zhao J., Zhao X., Shen X., Zhang Y., Zhang Y., Ye L., Li D., Zhu Q., Yin H. (2022). CircCCDC91 regulates chicken skeletal muscle development by sponging miR-15 family via activating IGF1-PI3K/AKT signaling pathway. Poult. Sci..

[77] Weng J., Zhang P., Yin X., Jiang B. (2018). The Whole Transcriptome Involved in Denervated Muscle Atrophy Following Peripheral Nerve Injury. Front. Mol. Neurosci..

[78] Reinoso-Sánchez J.F., Baroli G., Duranti G., Scaricamazza S., Sabatini S., Valle C., Morlando M., Casero R.A., Bozzoni I., Mariottini P. (2020). Emerging Role for Linear and Circular Spermine Oxidase RNAs in Skeletal Muscle Physiopathology. Int. J. Mol. Sci..

[79] Choe N., Jeong A., Joung H., Jeong D., Kim Y.K., Kook H., Kwon D.H. (2025). Circular RNA circAtxn10 regulates skeletal muscle cell differentiation by targeting miR-143-3p and Chrna1. Korean J. Physiol. Pharmacol..

[80] Zhang R., Feng W., Yang Y., Pan Y., Zou C., Wang L., Zhang S., Zhao Y., Wu Y., Wang J. (2026). The Evolutionarily Conserved TPM1 Super-Enhancer Drives Skeletal Muscle Regeneration via Mechanotransduction Signaling. Adv. Sci..

[81] Haque S., Ames R.M., Moore K., Pilling L.C., Peters L.L., Bandinelli S., Ferrucci L., Harries L.W. (2020). circRNAs expressed in human peripheral blood are associated with human aging phenotypes, cellular senescence and mouse lifespan. Geroscience.

[82] Fitz N.F., Kumar A., Su Y., Sharma M., Singh S., Koldamova R., Lefterov I., Sahu A., Ambrosio F., Rosano C. (2026). Serum-Derived Extracellular Vesicles as Biological Indicator of Mobility Resilience in Older Adults. Aging Cell.

[83] Meinecke A., Mitzka S., Just A., Cushman S., Stojanović S.D., Xiao K., Mooren F.C., Fiedler J., Thum T. (2020). Cardiac endurance training alters plasma profiles of circular RNA MBOAT2. Am. J. Physiol. Heart Circ. Physiol..

[84] Li B., Chen L., Su Y., Meng J., Su Z. (2026). The role of circular RNAs in mediating the protective effects of exercise against muscle degeneration and aging. Biogerontology.

[85] Shen Y., Shi Q., Nong K., Li S., Yue J., Huang J., Dong B., Beauchamp M., Hao Q. (2023). Exercise for sarcopenia in older people: A systematic review and network meta-analysis. J. Cachexia Sarcopenia Muscle.

[86] Whaikid P., Piaseu N. (2024). The effectiveness of protein supplementation combined with resistance exercise programs among community-dwelling older adults with sarcopenia: A systematic review and meta-analysis. Epidemiol. Health.

[87] Choi M., Kim H.Y., Bae J. (2021). Does the combination of resistance training and a nutritional intervention have a synergic effect on muscle mass, strength, and physical function in older adults? A systematic review and meta-analysis. BMC Geriatr..

[88] Liang J., Zhang H., Zeng Z., Lv J., Huang J., Wu X., Wang M., Xu J., Fan J., Chen N. (2023). MicroRNA profiling of different exercise interventions for alleviating skeletal muscle atrophy in naturally aging rats. J. Cachexia Sarcopenia Muscle.

[89] Unti M.J., Jaffrey S.R. (2024). Highly efficient cellular expression of circular mRNA enables prolonged protein expression. Cell Chem. Biol..

[90] ClinicalTrials.gov Study of Circular RNA Treatment in Patients with Radiation-Induced Xerostomia-1 (NCT06714253). NCT06714253.

[91] ClinicalTrials.gov Safety and Efficacy Study of RXIM002 in Severe, Relapsed or Refractory Autoimmune Diseases (NCT07322718). NCT07322718.

[92] ClinicalTrials.gov A Single Arm Clinical Study of Dendritic Cell Vaccine Loaded with Circular RNA Encoding Cryptic Peptide for Patients with HER2-Negative Advanced Breast Cancer (NCT06530082). NCT06530082.

[93] Juchem M., Cushman S., Lu D., Chatterjee S., Bär C., Thum T. (2024). Encapsulating In Vitro Transcribed circRNA into Lipid Nanoparticles Via Microfluidic Mixing. Methods Mol. Biol..

