# Supplementary material for: Circular RNAs in Skeletal Muscle Aging and Sarcopenia: Mechanistic Evidence, Biomarker Potential, and Therapeutic Readiness
Source: Int J Mol Sci. 2026 Oct 3;27(19):8844. doi: 10.3390/ijms27198844 (PMC13665384; doi:10.3390/ijms27198844)
Supplement: Supplementary file 1 [file ijms-27-08844-s001.zip › ijms-4596659-supplementary.pdf]

Supplementary Table S1

Molecular identity and independent modifiers of circRNA studies summarized in Tables 4 and 5

Candidate	Identity	Species and model	Human linkage	Intervention timing	Locus of action	Modality
circSnd1 [63]	SND1 host gene	Human muscle and myotubes; mouse atrophy models; C2C12	Aged-muscle association, n = 5 per age group; no phenotyped sarcopenia cohort	Varied; post-onset status not uniform	Muscle-intrinsic in mechanistic experiments	Endogenous target; gain/loss perturbation
circDdb1 [64]	DDB1 host gene; circDdb1-867aa	Human muscle; mouse atrophy models; C2C12	Five young and five aged samples; no phenotyped sarcopenia cohort	Varied across atrophy experiments	Muscle-intrinsic	Endogenous target; gain/loss perturbation
circFUT10 [27]	FUT10 host gene	Aged/adult mouse muscle stem cells; in vitro myogenic assays	None reported	No candidate-directed aged-organism intervention	Muscle stem cells	Endogenous target; in vitro gain/loss
circBBS9 [71]	BBS9-derived circRNA	Aged mouse muscle; C2C12 overexpression	None	Exercise exposure; cellular perturbation	Whole-muscle profiling and myotubes	Endogenous candidate; expression-based cell experiment
circNEB [72]	NEB host gene; circNEB-907aa	Bovine myoblasts; four 40-month-old rabbits; mouse/tree-shrew injury models	None	Local aged-rabbit administration; functional onset not documented	Muscle-localized	Vector-mediated circularizing precursor
circTmeff1 [65]	TMEFF1 host gene; TMEFF1-339aa	Mouse denervation, immobilization, inflammatory wasting; myogenic cells	None	Post-onset treatment reported	Muscle-intrinsic/localized	Endogenous target; inhibition/expression
Engineered circmiR-29b [73]	Designed circular miR-29b decoy	Mouse immobilization and other wasting models	None	Post-onset in established immobilization atrophy	Muscle-directed	DNA/vector-expressed decoy; not purified circRNA
circANAPC7 [60]	ANAPC7 host gene	Pancreatic-cancer cachexia and tumor cells	No sarcopenia linkage	Not a post-onset aging intervention	Systemic/tumor-mediated	Tumor-associated circRNA manipulation
circGLIS3 [58]	GLIS3 host gene	High-fat-diet mouse model; intramuscular adipogenesis	None	During metabolic model; post-onset efficacy unestablished	Muscle-quality/adipogenic compartment	Endogenous candidate perturbation
circAGO3 [75]	AGO3 host gene	Chicken inflammatory atrophy	None	Manipulation during induced inflammation	Muscle-intrinsic	Endogenous gain/loss perturbation
circCCDC91 [76]	CCDC91 host gene	Chicken myogenic systems; dexamethasone cell context	None	No candidate-directed aging intervention	Muscle cells	Predominantly in vitro perturbation
circSMOX / denervation profiles [77,78]	Profile-defined candidates; no common junction identity	Dexamethasone C2C12; ALS-related/denervated-muscle expression	No aged-human-muscle or clinical sarcopenia validation	Profiling/association	Mixed tissue/cell signals	Endogenous profiling candidates
circAtxn10 [79]	ATXN10 host gene	Human myogenic-cell discovery; C2C12 mechanism	Human myogenic-cell profiling; no aging/sarcopenia validation	No aged-organism intervention	Muscle cells	In vitro gain/loss
circTPM1 [80]	TPM1 host gene	Bovine cells; cross-species super-enhancer evidence; mouse regeneration	Conservation only; no human circRNA validation	No circRNA-specific in vivo intervention	Muscle-intrinsic	Bovine-cell circRNA perturbation; in vivo enhancer perturbation not circRNA-specific

Names follow the main manuscript. Genomic junction coordinates are not inferred where unavailable. Post-onset means treatment after phenotype establishment. Human linkage does not change the preclinical evidence level. Reference numbers match the revised manuscript. ALS, amyotrophic lateral sclerosis; circRNA, circular RNA.
